# High Spectral and Temporal Acuity in Primary Auditory Cortex of Awake Cats

**DOI:** 10.1007/s10162-023-00890-6

**Published:** 2023-02-16

**Authors:** John C. Middlebrooks, Lauren K. Javier-Tolentino, Akshat Arneja, Matthew L. Richardson

**Affiliations:** 1 Department of Otolaryngology, University of California at Irvine, D404 Medical Science D, Irvine, CA 92697-5310 USA; 2 Department of Neurobiology and Behavior, University of California at Irvine, Irvine, CA USA; 3 Department of Cognitive Sciences, University of California at Irvine, Irvine, CA USA; 4 Center for Hearing Research, University of California at Irvine, Irvine, CA USA

**Keywords:** Auditory cortex, Frequency response area, Tuning curve, Phase locking, Non-synchronized, Temporal representation, Non-monotonic, Auditory filters

## Abstract

Most accounts of single- and multi-unit responses in auditory cortex under anesthetized conditions have emphasized V-shaped frequency tuning curves and low-pass sensitivity to rates of repeated sounds. In contrast, single-unit recordings in awake marmosets also show I-shaped and O-shaped response areas having restricted tuning to frequency and (for O units) sound level. That preparation also demonstrates synchrony to moderate click rates and representation of higher click rates by spike rates of non-synchronized tonic responses, neither of which are commonly seen in anesthetized conditions. The spectral and temporal representation observed in the marmoset might reflect special adaptations of that species, might be due to single- rather than multi-unit recording, or might indicate characteristics of awake-versus-anesthetized recording conditions. We studied spectral and temporal representation in the primary auditory cortex of alert cats. We observed V-, I-, and O-shaped response areas like those demonstrated in awake marmosets. Neurons could synchronize to click trains at rates about an octave higher than is usually seen with anesthesia. Representations of click rates by rates of non-synchronized tonic responses exhibited dynamic ranges that covered the entire range of tested click rates. The observation of these spectral and temporal representations in cats demonstrates that they are not unique to primates and, indeed, might be widespread among mammalian species. Moreover, we observed no significant difference in stimulus representation between single- and multi-unit recordings. It appears that the principal factor that has hindered observations of high spectral and temporal acuity in the auditory cortex has been the use of general anesthesia.

## Introduction

Basic neural representation of sound spectra and timing in the primary auditory cortex (area A1) has been studied mostly in anesthetized animals. Generally, frequency tuning in those conditions is broader and temporal synchrony is limited to lower rates than one might expect based on perceptual results. The responses of a neuron to pure tones of varying frequency and sound level are given by its frequency response area (FRA). Neurons in area A1 of anesthetized animals typically exhibit V-shaped FRAs that show sharp frequency tuning at low sound levels but, at moderate levels, broaden considerably to between 0.5 and > 2 octaves (e.g., cat: [1], ferret: [2], squirrel monkey: [3], marmoset: [4], rat: [5], gerbil: [6], guinea pig: [7, 8]). In anesthetized conditions, synchrony of cortical spikes to repeated sounds or to modulation envelopes is limited to rates substantially lower than is seen at sub-cortical levels. For instance, many neurons in the inferior colliculus of anesthetized cats synchronize to modulation envelopes higher than 100 Hz [9], whereas phase-locking of cortical neurons is rarely observed at rates higher than ~ 25 clicks per second (cps), and mostly is limited to rates of ≤ 15 cps (click trains: [10–12], trains of tone pips: [13].

Reports of area A1 of awake marmosets have emphasized distinctive spectral and temporal sensitivity that is not commonly reported in other species under anesthesia, particularly not in anesthetized cats. In addition to V-shaped FRAs, neurons in area A1 of awake marmosets exhibit I-shaped and O-shaped FRAs [14, 15]. The I-shaped FRAs maintain a narrow, relatively constant frequency bandwidth across a wide range of sound levels. The O-shaped FRAs are restricted in both frequency and sound level. Cortical neurons in A1 of awake marmosets also can show tonic responses to tones throughout the duration of a sound, in contrast to the typically phasic responses in anesthetized cats (e.g., [16, 17]). Unlike cortical neurons in anesthetized cats, some marmoset neurons can phase lock to trains of unmodulated clicks at rates up to > 50 cps, and other neurons can represent the rates of click trains with non-synchronized tonic spike trains that increase in spike rate with increasing click rates up to 330 cps [18]. In anesthetized cats, there is some evidence of representation of click rates by the spike rates of just the onset responses of cortical neurons [12], but there is little evidence in cats of representation of click rates by the rates of tonic firing (for review, see [19]).

The distinctive sensitivity to sound spectra and timing seen in area A1 in awake marmosets might be adaptations for representing the rich vocal repertoire of that species. Alternatively, it has been suggested that the sharp spectral and level tuning that has been seen with single-unit recording in marmosets might have been blurred in other studies by use of multi-unit recordings [15, 20]. Here, we tested a third hypothesis, that many of the features observed in the awake marmoset are not restricted to primates but have been obscured in previous studies of other species by use of general anesthesia. We studied neurons in area A1 of alert cats using chronically implanted electrode arrays. Pure-tone stimuli that were varied in frequency and level revealed V-, I-, and O-shaped FRAs like those seen in awake marmosets, often with frequency bandwidths narrower than 0.2 octaves. Similar FRA characteristics were recorded both in isolated single units and in undifferentiated clusters of 2 or more units. That observation suggests that neighboring neurons share common FRA shapes. Click trains varying in rate were used to assess temporal acuity. About half of the single and multiple units in our sample that responded to the click trains synchronized to click rates of 5 cps or higher. Half of those synchronized units exhibited phase locking to rates higher than 30 cps, some to rates > 200 cps. We also observed representation of all presented click rates, from 5 to 320 cps, by the spike rates of non-synchronized tonic responses. These results show that the remarkable features of spectral and temporal representation previously observed in the marmoset auditory cortex are not limited to primates and suggest that such features might be widespread among the auditory cortices of mammals when studied in awake conditions.

## Methods

### Overview

Extracellular spike activity was recorded with 32-site recording arrays chronically implanted in area A1 of 12 cats. Seven of the cats (2 female and 5 male) had been trained in an auditory psychophysical task (spatial stream segregation; [21] and Arneja, unpublished results). The results in the present report, however, were obtained entirely during non-task conditions. That is, the response pedal and automatic feeder were removed from the apparatus, and the cat was given small amounts of food at irregular intervals intended to keep it alert and oriented toward the center of the coordinate system.

### Animal Preparation

All procedures were conducted in accordance with the National Institute of Health guidelines and with the approval of the Institutional Animal Care and Use Committee for the University of California at Irvine. Domestic shorthair cats (*Felis catus*), 9 males and 3 females, were obtained from a breeding colony at the University of California at Davis. In each animal, a chronic 32-site recording array, described below, was implanted in the auditory cortex in the right hemisphere using conventional aseptic procedures with isoflurane anesthesia. The recording array was implanted on the crown of the middle ectosylvian gyrus, which is the usual location of the ~ 8 kHz representation in the area A1. We avoided electrode placements further rostral in A1, where best frequencies (BFs) are higher, because such placements might have strayed into the anterior auditory area (area AAF; [22]). We also avoided electrode placements in area A2, located ventral to A1 and characterized by broader frequency tuning [23], and avoided placements in the dorsal zone of A1, also characterized by broad frequency tuning and by thalamic inputs that differ from those of the main body of A1 [24]. After approximately 2 weeks of recovery, we began physiological recordings for a period of several weeks to several months.

### Experimental Apparatus, Stimulus Generation, and Data Acquisition

Experiments were conducted in a double-walled, sound-attenuating booth (Industrial Acoustics; inside dimensions: 2.6 × 2.6 × 2.5 m) that was lined with 60-mm-thick absorbent foam (SONEXone). A circular hoop, 1.2 m in radius, supported 8.4-cm-diameter two-way loudspeakers at a range of locations in the horizontal plane; the present study used only the speakers at 0° and left 40°, contralateral to the electrode placement in the animal’s right hemisphere. During recording, the animal sat or stood on a raised pedestal that was adjusted in height to center the cat’s head in the array of loudspeakers. A harness restrained the animal to the pedestal but allowed free movement of the limbs and head.

Stimulus generation and data acquisition used System III hardware from Tucker-Davis Technologies (TDT; Alachua, FL). Custom MATLAB software (Mathworks; Natick, MA) on a Windows-based computer controlled the stimulus presentation, acquired the neural waveforms, and allowed online monitoring of responses. Sounds were generated at a rate of 97,656 samples per second with 24-bit precision. Sounds were calibrated in the absence of the cat using a precision ½-inch microphone (ACO Pacific) positioned at the center of the chamber at the normal location of the animal’s head. Tones were calibrated using pure-tone bursts in 1/24-oct intervals. Broadband sounds were calibrated using Golay codes as probes [25]. The calibration procedure for broadband sounds yielded a 1029-tap finite-impulse-response correction filter for each speaker. The filters flattened and equalized the responses of the loudspeakers such that, for each loudspeaker, the standard deviation of the magnitude spectrum across the 0.2–25-kHz calibrated passband was < 1 dB. The responses rolled off by 10 dB from 25 to 40 kHz.

Extracellular neural spike activity was recorded with N-Form 32-site chronic recording arrays from Modular Bionics (Berkeley, CA). Each array consisted of four parallel shanks, each containing 8 recording sites. The four shanks either were all 3 mm in length, or two were 2.5 mm and two were 3.5 mm in length. The shanks were arranged on corners of a rectangle 0.8 mm or 1.0 mm in the medio-lateral dimension and 0.8 mm or 1.5 mm in the rostro-caudal dimension. Recording sites were spaced in 250-μm intervals along each shank. The sites consisted of the cut ends of 25-μm-diameter platinum wires that were coated in activated iridium. Waveforms were recorded simultaneously from 32 sites using high-impedance head-stages and multichannel amplifiers from TDT. Waveforms were filtered, digitized at a rate of 24,414 samples per second, displayed online, and stored to computer disk for offline analysis.

### Experimental Procedure

Frequency response areas (FRAs) were probed with pure tones. Tones pips were 100 ms in duration with 5-ms raised-cosine onset and offset ramps, presented at 600-ms onset-to-onset intervals from the contralateral 40° loudspeaker (11 cats; 88 units) or from the 0° loudspeaker (one cat; 10 units); no differences in responses to the two loudspeaker locations were evident. Study of the set of 32 recording sites in each animal began with a coarse estimate of FRAs using 1/3-oct steps of frequency and 10-dB steps of sound level. The frequency that yielded the strongest response at each recording site was taken as an initial estimate of that site’s BF. Once the range of BFs was acquired across all active sites, FRAs were measured at a higher resolution using tones typically ranging in frequency from 1 oct below to 1 oct above the estimated BF range in 1/6- or 1/12-oct steps and typically ranging in level from − 30 to 60 dB SPL in 5-dB steps. Each combination of frequency and level was presented once in a random order, and then again in a different order, and so on for a total of 10 presentations of each frequency/level combination.

The representation of rates of periodic sounds was tested with trains of clicks. The click trains were 1 s in duration, presented at onset-to-onset intervals of 1.5 s, at click rates of 5 to 320 cps in ½ oct intervals. The clicks were biphasic, 51 µs per phase, which yielded spectral peaks at ~ 7.3 and 28.8 kHz and spectral nulls at 19.6 and 39.1 kHz. Click trains were presented from 0 and/or contralateral 40° loudspeaker locations. Usually both locations were tested in separate blocks of trials and, for each unit, results are presented from the location that yielded the higher synchronization boundary. In cases in which synchronization boundaries were measured at both loudspeaker locations, there was no systematic difference between synchrony at the two locations (*df* = 55*, z* = 0.60*, P* = 0.55).

### Data Analysis

We aimed to compare spectro-temporal sensitivity in the auditory cortex of alert cats with that in previously reported studies of the alert marmoset [14, 15, 18]. For that reason, qualitative measures of responses were patterned largely after those described in those previous reports.

Distributions of neural spike data generally did not conform well to normal distributions. For that reason, non-parametric statistical tests (from the MATLAB Statistics Toolbox) were employed. Wilcoxon rank sum tests compared the medians of two distributions. Wilcoxon signed-rank tests were used for pair-wise comparisons of the difference of two equal-size samples. The two-sample Kolmogorov–Smirnov test tested whether two samples were from the same continuous distribution; the statistic from that test is denoted *ks2test*. The Bonferroni correction was applied to account for multiple comparisons.

Data analysis began with offline identification of neural action potentials, as described previously [26]. Responses were classified as well-isolated single units when they met the following criteria: (1) the histogram of spike amplitudes showed a peak that was distinct from other activity; (2) the waveform appearance was consistent across trials upon visual inspection; (3) most of the inter-spike intervals were > 1 ms. Multi-unit recordings consisted of spikes from two or more undifferentiated neurons. Except when stated otherwise, “unit” refers collectively to single- and multi-unit recordings. Single- or multi-unit responses to tones and/or click trains were recorded at 150 sites in 12 cats. Non-responsive sites were assumed to be in non-responsive cortical layers or outside of the targeted auditory area.

Frequency sensitivity was quantified by analysis of FRAs, which consisted of mean spike rates as a function of sound frequency and level; analysis of FRAs was limited to units that showed response rates significantly greater than the spontaneous rate to tones at one or more frequencies (Wilcoxon rank sum; *P* < 0.05). Prior to any analysis of FRA properties, the spontaneous rate was subtracted from the neural response rate and a threshold was imposed equal to 20% of the above-spontaneous maximum spike rate; this follows the procedure employed by Sadagopan and Wang [15], Sutter and Schreiner [27], Moshitch et al. [28], and other groups. Except when stated otherwise, analyses of FRAs were conducted over the time range of 10 to 110 ms after stimulus onset; that time window allowed for ~ 3.5-ms acoustic travel time for the 1.2-m distance from the sound source to the cat and a minimum latency for neural conduction from the ears to the cortex. The frequency selectivity in an FRA was based on the profiles of spike rates versus frequency at each of several sound levels. The BF for each unit was computed by, first, averaging the spike rate at each frequency across all suprathreshold levels. Then, a spike-rate-weighted frequency profile was computed by multiplying each frequency by the corresponding mean spike rate. The frequency profile (in units of kHz × spikes/s) was then summed across frequencies and divided by the sum of the spike rates (in spikes/s). The resulting spike-rate-weighted average of stimulus frequencies (in kHz) was taken as the BF. The best level then was found by, first, computing a mean sound level profile formed by the Hann-weighted mean of sound-level profiles at the BF and the 2 adjacent frequencies. The best level then was taken as the sound pressure level at the peak of that mean level profile. The level profile was further characterized by the monotonicity index, which was the spike rate at the highest tested sound level divided by the maximum spike rate at the best level. Monotonicity index values could range from 0, indicating no response to the highest level tested, to 1, indicating that the highest tested level was the best level.

Frequency selectivity of units was represented by the bandwidth computed from frequency contours measured at constant sound levels. The bandwidth at a particular sound level was evaluated by computing the area of the spike-rate-weighted frequency profile at that level, then forming the rectangle having the same area with height equal to the maximum spike rate, and taking the width of that rectangle, expressed in octaves. In instances at which there was no response at a particular sound level, the bandwidth at that level was denoted as 0 oct.

The time course of responses to tones was quantified by the sustained index, which was the spike count at the BF and best level in the second half of the tone duration (60–110 ms) divided by the spike count across the entire tone duration (10–110 ms); both ranges included an adjustment of 10 ms for a 3.5-ms acoustic travel time and for a minimum neural travel time from ear to cortex. Prior to the calculation of a sustained index, the mean spontaneous rate was subtracted from the response and a 20% threshold was imposed, identically to all other analyses of FRAs.

We evaluated the representation of the rates of periodic stimuli by quantifying the spike timing and spike rates of responses to click trains at various rates. Analysis of stimulus synchrony of spikes was based on the response interval from 100 to 1010 ms post stimulus onset; that time window excluded the response to the onset of the click train. For analysis of synchrony to each click train, each spike was treated as a unit vector oriented as the phase of the spike relative to the phase of the periodic click train. Vector strength was calculated by taking the sum of the unit vectors and dividing by the number of spikes [29]. Significance of spike synchrony was tested using the Rayleigh test of circular uniformity. The Rayleigh statistic was given by *2Nr*^*2*^ [30], where *r* is the vector strength and *N* is the number of spikes; a Rayleigh statistic of $\ge$ 13.8 indicates significant synchrony (*P* < 0.001). The criteria for significant vector strengths varied with the number of spikes, as *r* = $\sqrt{13.8/2N}$. The synchronization boundary of each unit was defined as the highest interpolated click rate at which the vector strength exceeded that criterion.

Many neurons that failed to synchronize to high click rates could represent those click rates with systematic increases in tonic spike rates. Analysis of click-rate representation by tonic spike rates was limited to 100 to 1010 ms relative to onset of the click train; this was intended to exclude responses to the first few clicks of the train, which often were insensitive to the rates of ensuing clicks. A Kruskal–Wallis test assessed the dependence of spike rates on click rates. When that test was significant, a Bonferroni-corrected post hoc analysis tested for significant increases in spike rates elicited by pairs of increasing click rates; non-monotonic patterns that included decreases in spike rate for increasing click rates were also observed but significant decreases in spike rate were far less common than significant increases. The lower boundary of the dynamic range of spike-rate representation by each unit was the lowest click rate at which an increase in click rate elicited a significant increase in spike rate. The upper boundary of the dynamic range was at the nearest peak of the profile of spike rate versus click rate above the lower boundary. The breadth of the dynamic range was given by the ratio (in octaves) of click rates between the upper and lower boundaries of the range. The depth of spike-rate modulation by click rates was given by the maximum minus minimum spike rates within the dynamic range of click rates, expressed as a percentage of the maximum minus minimum spike rates across all click rates.

## Results

### Spectral Sensitivity

Spectral response properties were measured using pure tone pips, 100 ms in duration and varying in frequency and sound level. A total of 103 units responded significantly to such stimuli in 12 alert cats. Of those 103 units, 16 well-isolated single units and 82 multi-unit sites (total = 98) yielded FRAs that could be classified as V/I or O, as described below; the remaining 5 FRAs were too patchy to classify (Table 1).

**Table 1:** Numbers of units in the present study and in Sadagopan and Wang [15] classified by single- and multi-unit spike isolation and by O and V/I FRA types

		O units	V/I units	Other	Total tone-responsive
Present study, awake cat	Well-isolated single units	5	11	3	19
	Multi-unit sites	19	63	2	84
	Total	24	74	5	103
Sadagopan and Wang [15], awake marmoset	Well-isolated single units	175	100	39	314

Sadagopan and Wang [15] reported that all studied units were well isolated. “Other” indicates patchy or otherwise non-classifiable FRAs

**Fig. 1:**
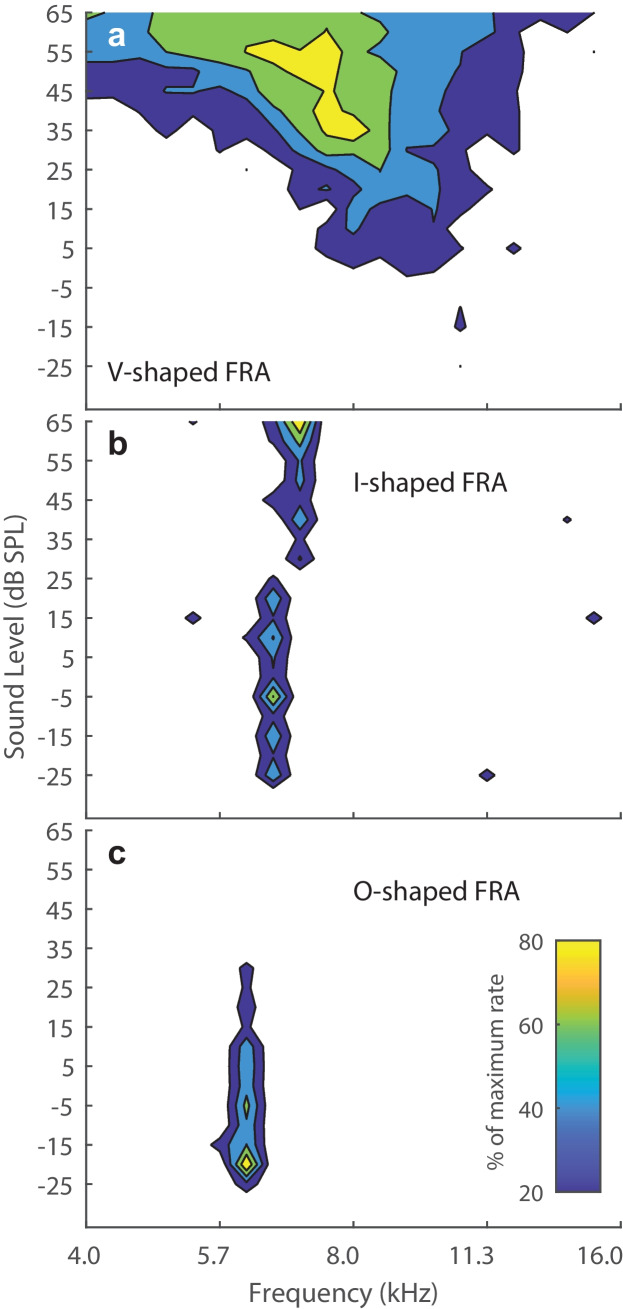
Frequency response areas (FRAs) of three units recorded in one animal. In each panel, sound levels are shown on the vertical axis, tone frequencies are shown on the horizontal axis, and mean spike rates as percentages of the maximum rate for each unit are represented by colors. (**a**) V-shaped FRA from a well-isolated single unit. The BF and best level were 7.8 kHz and 35 dB SPL. (**b**) I-shaped FRA from a multi-unit recording. The BF and best level were 6.5 kHz and − 5 dB SPL. (**c**) O-shaped FRA from a multi-unit recording. The BF and best level were 6.1 kHz and − 20 dB SPL

Neurons exhibited several distinct types of FRAs. We classified unit types as V, I, and O based on visual inspection of their FRAs (following the study in the inferior colliculus by [31]. That qualitative classification corresponded well with quantitative measures, which are presented below. Representative examples of FRAs from one animal are shown as contour plots in Fig. 1. The V units (as in Fig. 1(a)) showed their narrowest frequency tuning at the lowest suprathreshold sound levels. The response magnitude and breadth of their tuning generally increased as sound levels were increased. The I units (e.g., Fig. 1(b)) showed narrow frequency tuning that was essentially constant in bandwidth with increasing sound levels above threshold; often there was a slight shift in the sensitive frequency band at high sound levels as in this example. Across the sample, both V and I units maintained responses at the highest tested sound levels. Bandwidths at the highest sound levels were distributed continuously from those of I to those of V units, which precluded a quantitative distinction between those unit types. For that reason, V and I units are denoted hereafter as “V/I.” The O units (e.g., Fig. 1(c)) were characterized by strong response magnitude and sharp frequency tuning at a best level and by a response that weakened markedly or disappeared at higher sound levels. The non-monotonic response magnitude as a function of increasing sound level was the principal defining feature of O units. Our sample consisted of 24 O and 74 V/I units (Table 1). We encountered both V/I and O units in 7 of the 12 cats, whereas we encountered only V/I units in the other 5 cats; the cats failing to show O units generally had only limited samples of units showing significant responses to tones.

**Fig. 2:**
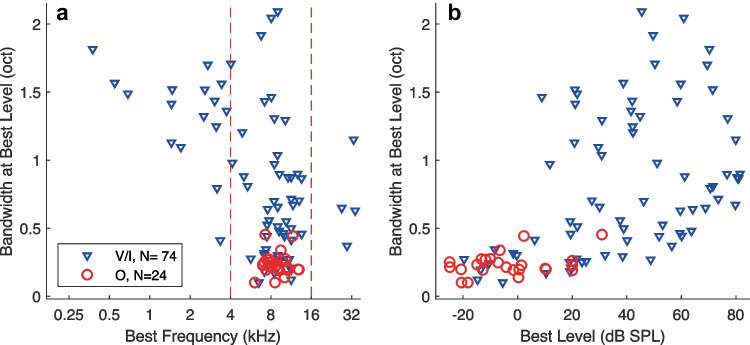
(**a**) Relationship between bandwidth at best level (vertical axis) and best frequency (horizontal axis). Dashed vertical lines indicate the range of best frequencies within ± 1 oct of 8 kHz. O and V/I units are distinguished by symbol shapes and colors, as indicated. (**b**) Bandwidth at best level versus best level. Values are jittered in the horizontal (best level) dimension to improve visibility

The BFs of our sample of units were largely concentrated within an octave of 8 kHz (Fig. 2(a); V/I and O units are denoted by distinct symbol types). Across all 98 O and V/I units, the median BF was 8.5 kHz, and 78 units had BFs between 4 and 16 kHz. The distribution of BFs reflects our placement of recording probes on the crown of the ectosylvian gyrus, which is the typical location of the ~ 8-kHz representation in the area-A1 tonotopic map (e.g., [28, 32–34]) different placements would have yielded different BF distributions. Our BF sample coincides well with some other studies of frequency tuning in the cat’s area A1. Table 2 gives summary values of spectral representation in A1 of cats and marmosets in various alert and anesthetized conditions.

**Table 2:** Spectral sensitivity in area A1 of cats and marmosets

	Species, anesthetic condition	CF or BF	Bandwidth	Bandwidth near 8 kHz	Monotonicity Index	% non-monotonic units
**a**	Cat, awake	BF, median = 8.5 kHz; geometric mean = 7.1 kHz; mostly 4 to 16 kHz	BW at best level, median: All units = 0.47 oct O units = 0.22 oct V/I units = 0.70 oct	BW at best level, median: all units = 0.34 oct O units = 0.22 oct V/I units = 0.54 oct	Mean = 0.67	27%, 56%
**b**	Cat, barbiturate	CF mostly 5 to 25 kHz	BW40 mean = 0.68 oct		Mean = 0.71	23%, 53%
**c**	Cat, halothane	CF geometric mean = 6 kHz	Mean BW40 = 4 oct		Mean = 0.8	38%
**d**	Marmoset, awake	BF median = ~ 4 kHz	BW at best level, median: All units ~ 0.35 oct O units = 0.25 oct V/I units = 0.52 oct			64%
**e**	Marmoset, barbiturate	CF median = ~ 7 kHz	BW40 median = 0.6 to 1.4	Median = ~ 0.7 oct		
**f**	Marmoset, sufentanil	CF	BW20 mean = 1.0		Mean = 0.74	46%

**Fig. 3:**
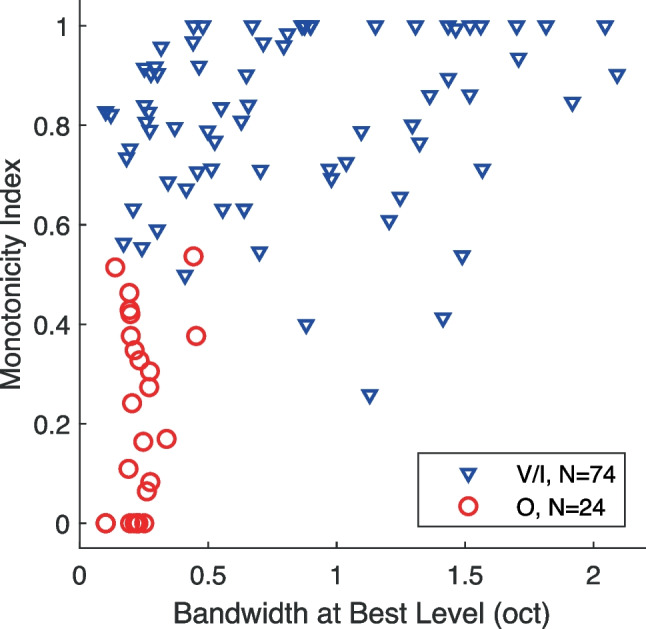
Relationship between monotonicity index (vertical axis) and bandwidth at best level (horizontal axis). There was a significant correlation between monotonicity index and bandwidth at best level (*r* = 0.48, *P* < 10^−6^)

**Fig. 4:**
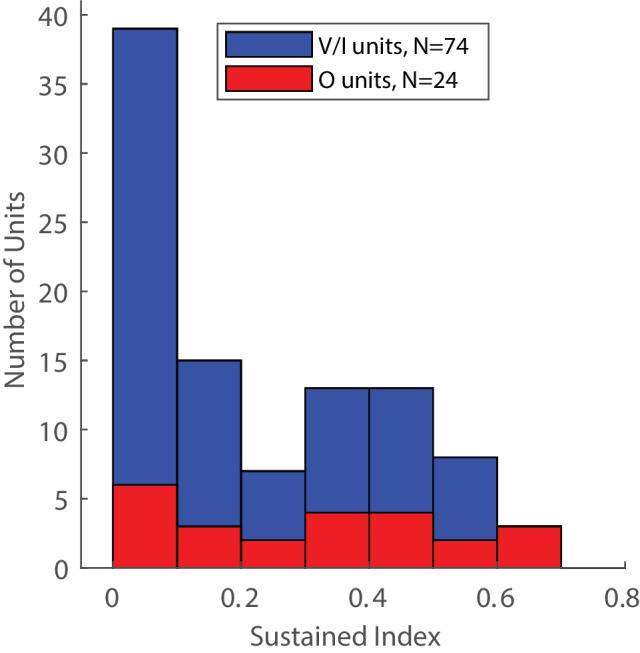
Distribution of sustained indices (as defined in Results). Numbers of V/I and O units are distinguished by colors, as indicated

Empty cells correspond to values that were not available in the publications. Bandwidths of units, in octaves, were measured at best level or 20 dB or 40 dB above unit threshold (denoted BW20 or BW40, respectively). Page and table numbers refer to those of the cited references. **Row a** (the present study): Values are as described in the main text. Bandwidth near 8 kHz is from units with BF between 4 and 16 kHz. % non-monotonic units is the percentage of units with monotonicity index ≤ 0.50 and ≤ 0.80, respectively. **Row b** [20, 34]: Characteristic frequency (CF) is estimated from inspection of Fig. 2 of the 1992 paper Schreiner and Sutter [34]. BW40 is the weighted mean of single- and multiple-unit means in Table 1. Monotonicity index is the unit-count-weighted mean measured from Fig. 6b of the 1995 paper Sutter and Schreiner [20]. The % non-monotonic units is the percentage of single units with monotonicity ≤ 0.50 and ≤ 0.80, respectively, estimated from Fig. 6b. **Row c** [28]: The % non-monotonic units is for monotonicity index ≤ 0.80. **Row d** [15]: BF estimated from Fig. 2(e). Bandwidth is the unit-count-weighted mean of medians of O and V/I units given in Fig. 3(a). The % non-monotonic units is for MI ≤ 0.75. **Row e:** [35]: Median CF is estimated from Fig. 3. The bandwidth was estimated from the range of Q40 across CFs shown in Fig. 4(e, f) and then expressed in octaves. The bandwidth around 8 kHz was estimated from the Q40 around CF of 8 kHz and then expressed in octaves. **Row f** [36]: The bandwidth was the mean across cortical layers (pg. 931). The monotonicity index was the mean across layers weighted by unit counts studied in various layers (pg. 929). The % non-monotonic units (monotonicity index ≤ 0.80) was across all layers

We represented the frequency selectivity of each unit by its bandwidth, in octaves, computed at its best level (Fig. 2(b)); see Methods for the computation of best level and bandwidth. Across all O and V/I units, the median bandwidth at best level was 0.47 oct. All the O units in our sample had bandwidths < 0.5 oct. The distribution of bandwidths of V/I units was more widespread. The narrowest bandwidths were similar between O and V/I units, whereas the V/I bandwidths could be as high as ~ 2 oct; the distributions of O and V/I bandwidths were significantly different (*ks2stat* = 0.71, *P* < 10^−7^). The narrowest bandwidths in our sample were among units having BFs within an octave of 8 kHz. Some previous studies have characterized frequency selectivity by the bandwidth at 40 dB above units’ thresholds (e.g., [34]). In our sample, there was no significant difference between the distributions of bandwidths at best level compared with bandwidths at 40 dB above thresholds (*ks2stat* = 0.11, *P* = 0.54; *Wilcoxon signed rank test of paired bandwidths* = 2116, *z* = 1.91, *P* = 0.055).

Across all units, the sharpest frequency tuning generally belonged to units having the lowest best levels (Fig. 2(b)). Among the 45 units with best levels ≤ 20 dB SPL, the median bandwidth at best level was 0.25 oct. That range of best levels included all but one of the O units (median bandwidth of all O units: 0.22 oct). That means that the O units, and many of the V/I units showing narrowest bandwidths, are largely inactive at moderate-to-high sound levels of, say, ≥ 40 dB SPL, which are common sound levels for everyday listening. The distribution of best levels of V/I units reached nearly as low as that of O units but extended to the highest sound levels that were tested. Among the 42 units having best levels at or above ≥ 40 dB SPL, all of which were V/I units, the median bandwidth at best level was 0.88 oct. That is, cortical neurons that showed their strongest responses at moderate sound levels had a median bandwidth more than three times as broad as that of neurons that responded most strongly at low sound levels. In contrast to that trend, we note that human listeners show more acute frequency discrimination at moderate-to-high sensation levels than at lower levels (e.g., [37]). As a point of reference, we recently estimated the Equivalent Rectangular Bandwidth of the cat’s auditory filter using a psychophysical notched-noise procedure [38]: the value of the filter centered at 8 kHz was 0.2 oct. Of the 78 units in our sample having BFs within an octave of 8 kHz, only 14 (18%) had bandwidths ≤ 0.2 oct, and all those units had best levels ≤ 20 dB SPL.

The bandwidths of V/I units broadened with increasing sound level across the entire tested range of levels, more so for V than for I units. We computed bandwidths and BFs of V/I units at sound levels of 20, 40, and 60 dB SPL; O units were excluded from that analysis because many O units show marked decreases in responses across those sound levels. Median bandwidths of V/I units measured at the three levels were 0.46, 0.62, and 0.69 oct, respectively, which is a significant broadening of bandwidths across those levels (analysis of variance: *F*(2,134) = 42, *P* < 10^−7^). Nevertheless, broadening in bandwidth was not uniformly distributed across V/I units. Bandwidths of half of the V/I units expanded by no more than 0.2 oct at levels from 20 to 60 dB SPL. Those units, therefore, appeared more I-like than V-like in that their bandwidths were fairly constant across a 40-dB range of stimulus levels. Among the units that showed the greatest broadening of bandwidths across increasing sound levels, the FRAs tended to expand on the low-frequency side, as indicated by shifts in BF to lower frequencies; the unit in Fig. 1(a), for example, showed a shift in BF from 8.5 kHz at 20 dB SPL to 6.9 kHz at 60 dB SPL. The half of V/I units showing bandwidth expansions > 0.2 oct showed a median shift in BF of 0.19 oct downward, whereas units showing expansion < 0.2 oct showed a negligible upward shift in BF of 0.013 oct. There was a significant negative correlation of shift in BF (in octaves) with broadening of bandwidth (in octaves): *r* =  − 0.29, *P* = 0.015).

The qualitative classification of FRAs of O and V/I units by visual inspection coincided closely with quantitative ranges of monotonicity indices (Fig. 3). The monotonicity index is the ratio of the response rate of a unit at the maximum tested sound level divided by the response rate at its best level (see the “Methods” section). Sadagopan and Wang [15] used a monotonicity-index criterion of 0.75 to designate non-monotonic units, whereas some other studies have used a criterion of 0.80 for non-monotonic or 0.50 for “strongly non-monotonic” [17, 20]. In our sample, 56% of units had monotonicity indices ≤ 0.80, and 27% had indices ≤ 0.50. All but 2 of the units in our sample that were classified qualitatively as having O-shaped FRAs had monotonicity indices ≤ 0.5 (median across all O units = 0.21). Seven O units had monotonicity indices of zero, indicating that they showed no response to the highest tested sound levels. The V/I units generally had higher monotonicity indices (V/I median = 0.83). Three units had monotonicity values < 0.5 but bandwidths > 0.5 oct—those were judged to be V units that showed somewhat patchy FRAs. The observed differences in monotonicity between O and V/I units were to be expected, as non-monotonic level sensitivity was a defining characteristic of O units.

Most units in our sample included spikes from more than one neuron. Of the 98 units for which FRAs were analyzed, 16 were well-isolated single units. Of those single units, ~ 31% (5/16) displayed O-shaped FRAs, compared to ~ 23% of multi-unit sites (19/82). Single unit sites did not differ significantly from multi-unit sites in their bandwidth at best level (*z* = 0.16, *P* = 0.87), and medians of monotonicity index values were only slightly lower for single- than for multi-unit sites (0.65 versus 0.79, *z* = 2.0, *P* = 0.048).

### Tonic Responses to Tones

We observed a variety of temporal responses to tones ranging from phasic (i.e., limited to near the tone onset) to tonic responses that persisted throughout the tone duration. We quantified temporal response patterns with a sustained index, which was defined as the spike count in the latter half of the tone duration (60–110 ms after tone onset) divided by the spike count across the entire tone duration (10 ms–110 ms); those time ranges include 10 ms for acoustic travel time and ear-to-cortex latency. A sustained index of zero would indicate no spikes in the latter half of the stimulus duration (indicative of phasic responses), and sustained index value of 0.5 would indicate equal spike rates throughout initial and latter halves of the 100-ms stimulus (indicative of tonic responses); a few units with sustained indices > 0.5 were those that had somewhat long onset latencies and, hence, reduced spike counts during the initial half of the stimulus. The sustained index was computed for responses at each unit’s BF and best level. Figure 4 displays the distribution of sustained indices for V/I- and O-shaped FRAs. A large proportion of V/I units exhibited phasic responses, with near-zero sustained indices (median across all V/I units = 0.14), whereas the sustained indices of O units were more uniformly distributed (median = .37) and included a larger proportion of tonic responses; median sustained indices were lower among the V/I units (*ranksum* = 3375, *P* = 0.015) although the difference in distributions of sustained indices for O versus V/I units was not significant (*ks2stat* = 0.30, *P* = 0.065).

**Fig. 5:**
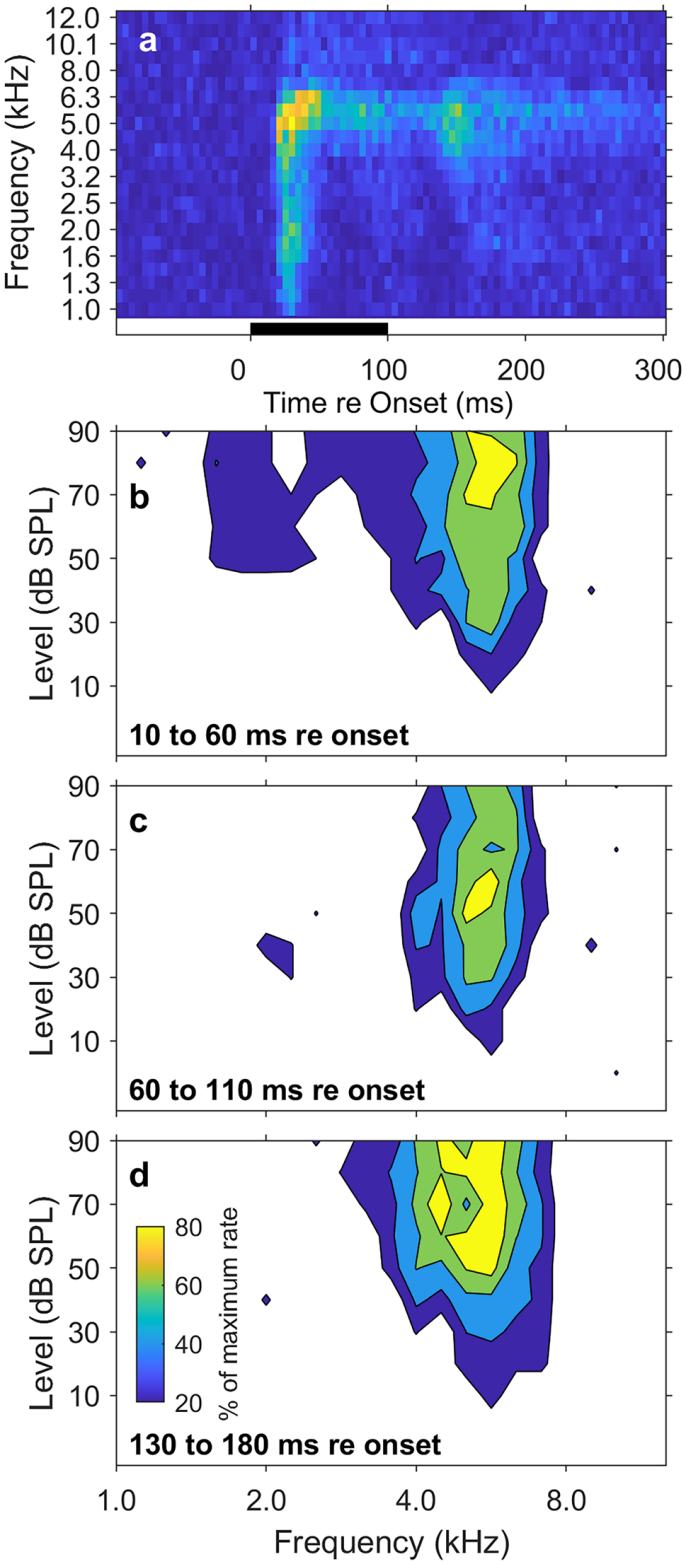
(**a**) Post-stimulus-time histograms (PSTHs) for one unit. Each horizontal row of pixels represents a PSTH at a single frequency indicated on the vertical axis; in each row, responses are collapsed across sound levels from 40 to 90 dB SPL. Mean spike rates are represented by colors, with warmer colors denoting higher spike rates. The black bar at the bottom of the plot indicates the tone duration. (**b**, **c**, **d**) FRAs of the same unit computed for various time ranges re tone onset, as indicated in each panel. Colors indicate mean spike rates as percentages of the maximum rate for each time range. The color scale in panel d applies to panels **b**, **c**, and **d**

Inspection of post-stimulus-time histograms (PSTHs) suggested that, in many instances, the frequency tuning of units narrowed with increasing time after stimulus onset, such that sustained responses were restricted to frequencies close to units’ BFs. That property can be seen in the example V/I unit represented in Fig. 5. Figure 5(a) shows PSTHs, with each row of pixels representing a PSTH at a single frequency. The sustained index of this unit at its best level was 0.33. The frequency range of the onset response of this unit was quite broad (bandwidth at best level = 1.0 oct), narrowing to 0.56 oct during the latter half of the tone, and then expanding somewhat to 0.79 oct during a late response, 30 to 80 ms after tone offset. The sharpening of bandwidth can be observed in contour plots of FRAs from this unit computed during 3 50-ms time ranges relative to tone onset: 10 to 60 ms (Fig. 5(b)); 60 to 110 ms (Fig. 5(c)); and 130 to 180 ms (Fig. 5(d)). Again, the frequency tuning was relatively broad early in the tone duration and sharpened later in the tone duration.

**Fig. 6:**
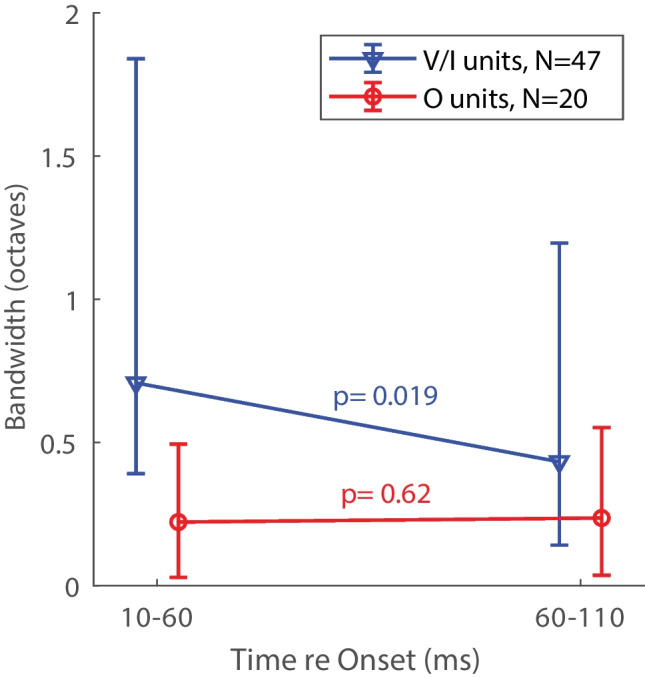
Bandwidths of units exhibiting sustained responses computed for early (10–60 ms) and late (60–110) late times after tone onset. The analysis is limited to the units having non-zero sustained indices: *N* = 47 V/I units and *N* = 20 O units. Lines and bars denote medians and quartiles. The *P* values are from rank sum tests

The narrowing of frequency tuning with increasing post-onset time was limited largely to the V/I units. Figure 6 illustrates bandwidths that were computed early (10–60 ms) and late (60–110 ms) after tone onset; the error bars show the quartiles. The analysis is limited to the 67/98 of units having non-zero sustained indices. Across the population of V/I units having sustained responses, bandwidths decreased by 39% in the latter part of the response; median bandwidths of V/I units were 0.71 oct for the first and 0.43 oct for the latter halves of the tone duration (*z* = 2.4, *P* = 0.019). Sustained responses were somewhat more common among O units, but bandwidths of O units tended to remain essentially unchanged between the first and latter halves of the 100-ms tone bursts (median bandwidths = 0.22 and 0.24, respectively; *z* = 0.50, *P* = 0.62). The difference between late and early bandwidths was significantly greater for V/I than for O units (*z* = 3.3, *P* = .0011).

### Click-Rate Sensitivity

Neurons responded to various rates of periodic stimuli by explicit synchrony to click trains and/or by non-synchronized tonic responses that increased in spike rate with increasing click rates. We evaluated responses to click trains presented at rates from 5 to 320 cps in ½-oct steps. Click-train stimuli elicited responses from 25 well-isolated single units and from 125 multi-unit sites.

#### Synchronized Responses

**Fig. 7:**
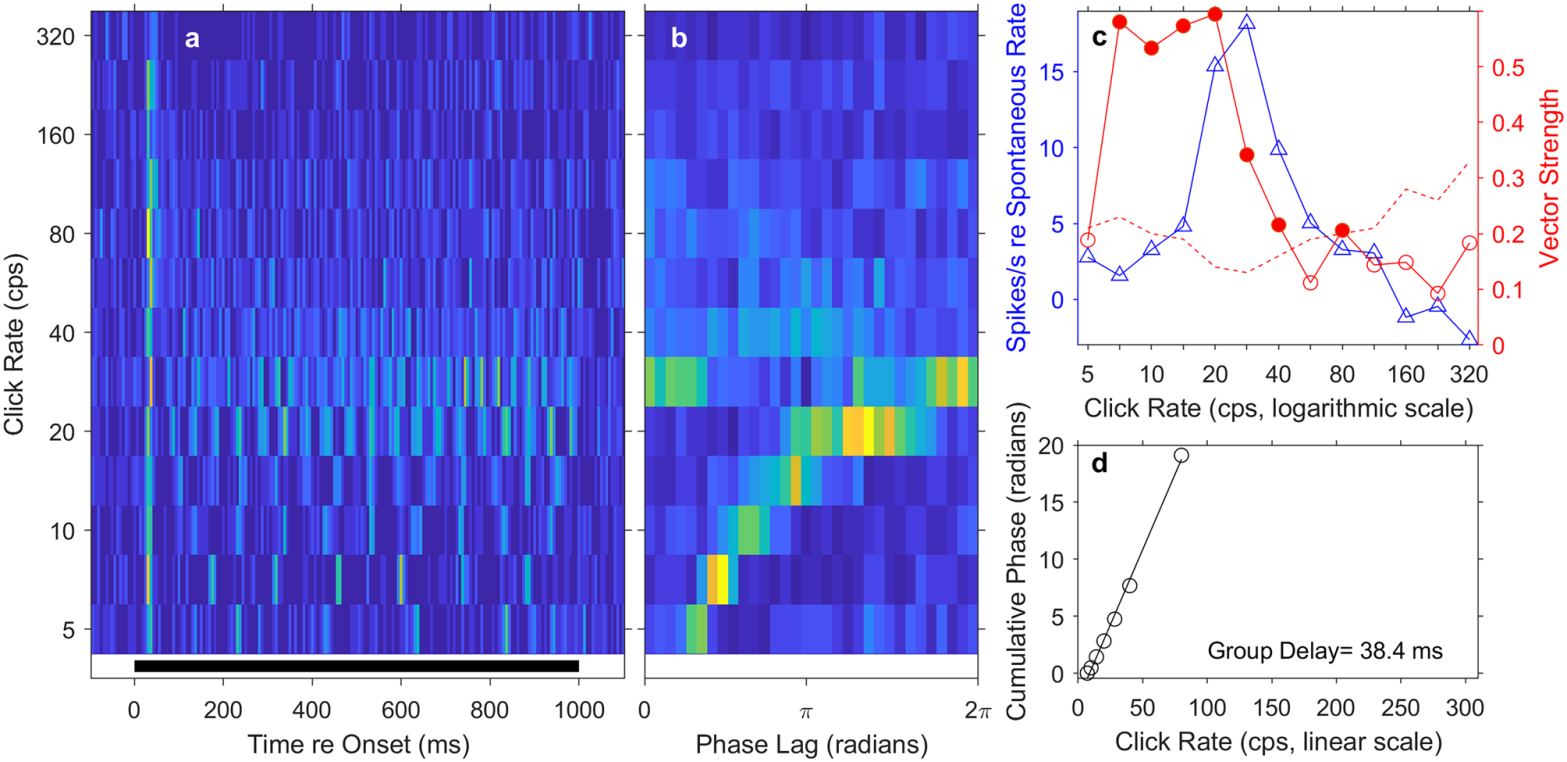
Temporal responses of a well-isolated single unit. (**a**) Each row of pixels represents a PSTH for a particular click rate indicated on the vertical axis. Mean spike rates are represented by colors, with warmer colors denoting higher spike rates. The black bar at the bottom of the panel indicates the duration of the click trains. (**b**) Each row of pixels represents a period histogram for a particular click rate. Each period histogram shows mean responses to clicks expressed as phase lags relative to the repetition period of the click train. The analysis was limited to the range of 100 to 1010 ms after the onset of the click train. (**c**) Summary measures plotted on a logarithmic scale of click rate. The blue solid line with triangles and the left vertical axis represent mean spike rates in the post-onset-range of 100 to 1010 ms as a function of click rate. Values are given as the mean spike rate minus the mean spontaneous rate for each unit. The red solid line with circles and the right vertical axis represent the vector strength, which is a measure of stimulus synchrony defined in the “Methods” section. The dashed red line indicates the criterion for significant phase locking. Vector strengths higher than the criterion (denoted by filled circles) were significant according to the Rayleigh test described in Methods (*P* < .001). (**d**) Cumulative phase lag, plotted on a linear scale of click rate. Data are plotted only for click rates at which phase locking was significant. The solid line plots the regression. The group delay of 38.4 ms was computed from the slope of the regression line

Significant synchrony to click rates 5 cps or higher was observed in 72 of the 150 units that responded to click trains; 12/72 were well-isolated single units, and 60/72 were multi-unit sites. Figure 7 shows the responses of a synchronized unit. Figure 7(a) plots the PSTH of this unit at each of the tested click rates, and Fig. 7(b) shows the corresponding period histograms. In the PSTHs (Fig. 7(a)), this unit displayed a prominent onset response at all click rates, followed by tonic stimulus-synchronized responses to click trains having rates up to 40 cps or higher. The period histograms (Fig. 7(b)) plot the mean spike rates of this unit expressed relative to the periods of the click trains. Accumulation of spikes at particular phase lags indicated phase-locked synchronous firing; peaks of the period histogram tended to broaden with increasing click rate, in part, because any given time duration (in ms) was mapped onto increasingly wide ranges of phase (in radians) as the periods of click trains shortened. Figure 7(c) plots the spike rates and vector strengths of the same unit on a logarithmic scale of click rate; measurements were restricted to the post-onset times of 100 ms to 1010 ms, which excluded the rate-insensitive onset response. The solid blue curve and left axis show the mean tonic driven spike rate as the measured spike rate minus the spontaneous firing rate; there was no 20% threshold applied as was done for analysis of FRAs. The tonic spike rate for this unit peaked at a click rate of 28 cps, with above-spontaneous tonic responses for click rates up to 113 cps. The solid red line and the right axis represent the vector strength, which is a measure of synchrony to the click trains (see the “Methods” section). The synchrony of this unit peaked at a click rate of 20 cps. The dashed red line indicates the criteria for significant vector strengths according to the Rayleigh test (*P* < 0.001*,* described in the “Methods” section); the criterion for significant vector strength increases with decreasing spike count. This unit showed significant synchrony (denoted by filled circles) up to an interpolated click rate of ~ 49.5 cps. Figure 7(d) plots the cumulative mean phase lag of the response as a function of click rate; only the points at which the vector strength was significant are shown. On this linear scale, the phase values trace a straight line, the slope of which yields in this case a group delay of 38.4 ms.

**Fig. 8:**
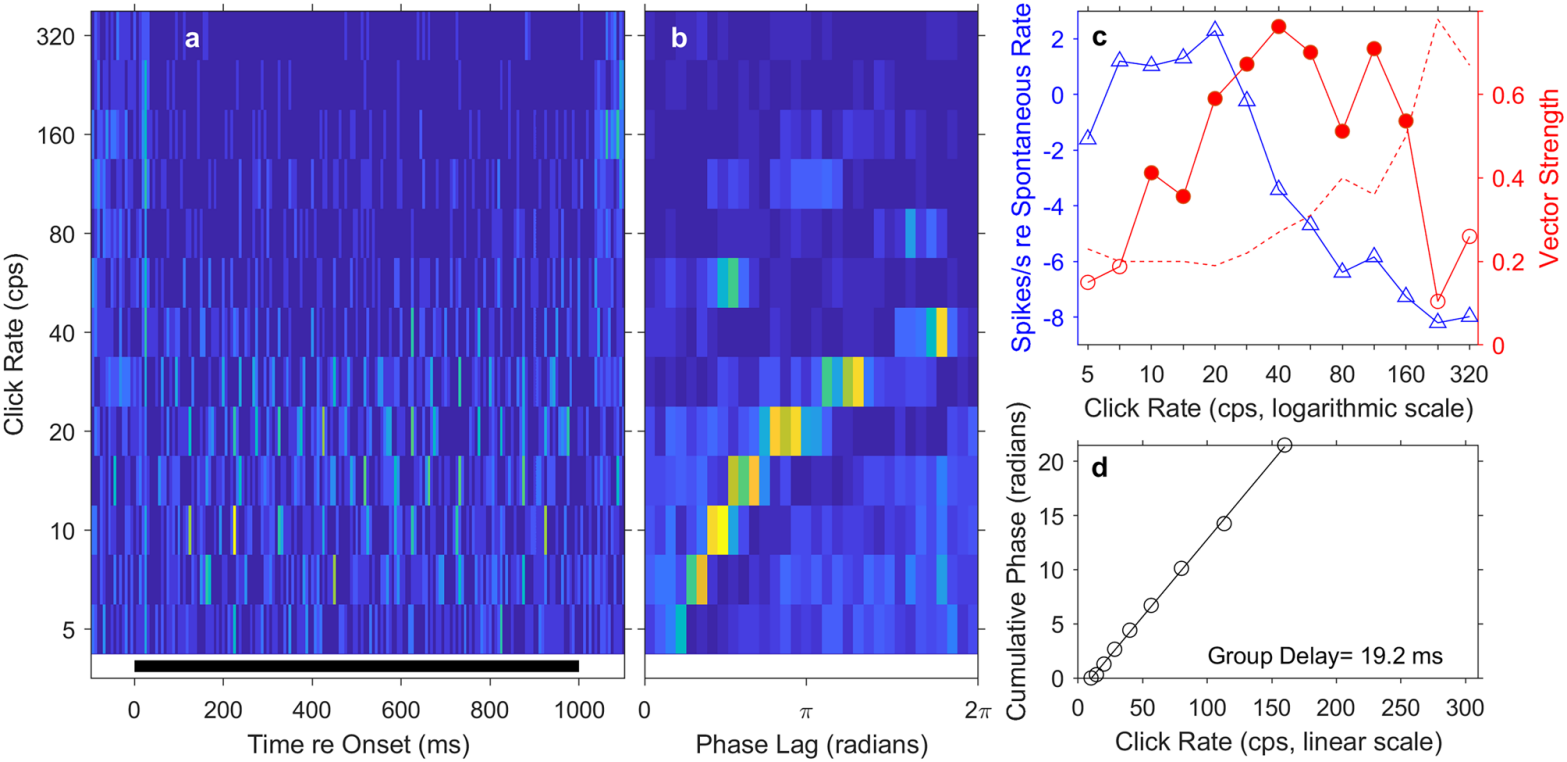
Temporal response of a well-isolated single unit, in this case showing phase locking at high click rates. Format of the 4 panels is identical to that in Fig. 7

Most of the synchronized units in our sample (63/72) synchronized to click rates no higher than 80 cps, but 9 units (1 single unit and 8 multi-unit sites) synchronized significantly to click rates as high as 140 to 309 cps; relevant to a point raised in the “Discussion” section, only 6 units synchronized significantly to click rates of > 188 cps. Figure 8 represents an example of a well-isolated single unit that synchronized to high rates. That unit showed peak synchrony at 40 cps, but its vector strength was significant for click rates up to 160 cps, with an interpolated synchronization boundary of 167 cps. The mean tonic spike rate of this unit was near the spontaneous rate at clicks rates up to about 28 cps and then dropped to well below the spontaneous rate at higher click rates. That the unit showed significant synchrony to the click train even when mean tonic spike rates were below spontaneous suggests that synchrony was accomplished by stimulus-locked inhibition of spontaneous firing. That is evident as bright peaks in the period histograms followed by suppressed background activity appearing as patches of dark blue (Fig. 8(b); particularly evident at click rates of 5–40 cps). The group delay of this unit was 19.2 ms, half that of the unit shown in Fig. 7.

**Fig. 9:**
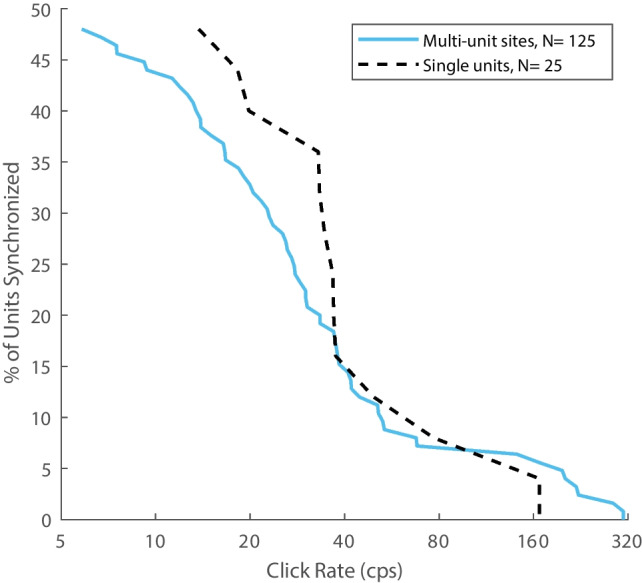
Cumulative distribution of synchronized units. The curves trace the percentage of multi-unit sites (blue lines) or well-isolated single units (dashed black lines) that were synchronized at each click rate (given on the horizontal axis). The unit counts (*N*) values are the counts of units that responded in any way to the click trains

The percentages of units that synchronized to each click rate are illustrated in Fig. 9 for well-isolated single units (dashed black curve) and multi-unit sites (solid blue curve); values are percentages of the 125 multiple or 25 single units that gave spike responses to click rates. Among the 72 units that synchronized to click rates ≥ 5 cps, median synchronization boundaries were 36 cps for single units and 28 cps for multi-unit sites. Single- and multi-unit sites did not differ significantly in the distributions of their synchronization boundaries (*ks2stat* = 0.16, *P* = 0.62).

#### Suppression of Tonic Responses and Offset Responses

**Fig. 10:**
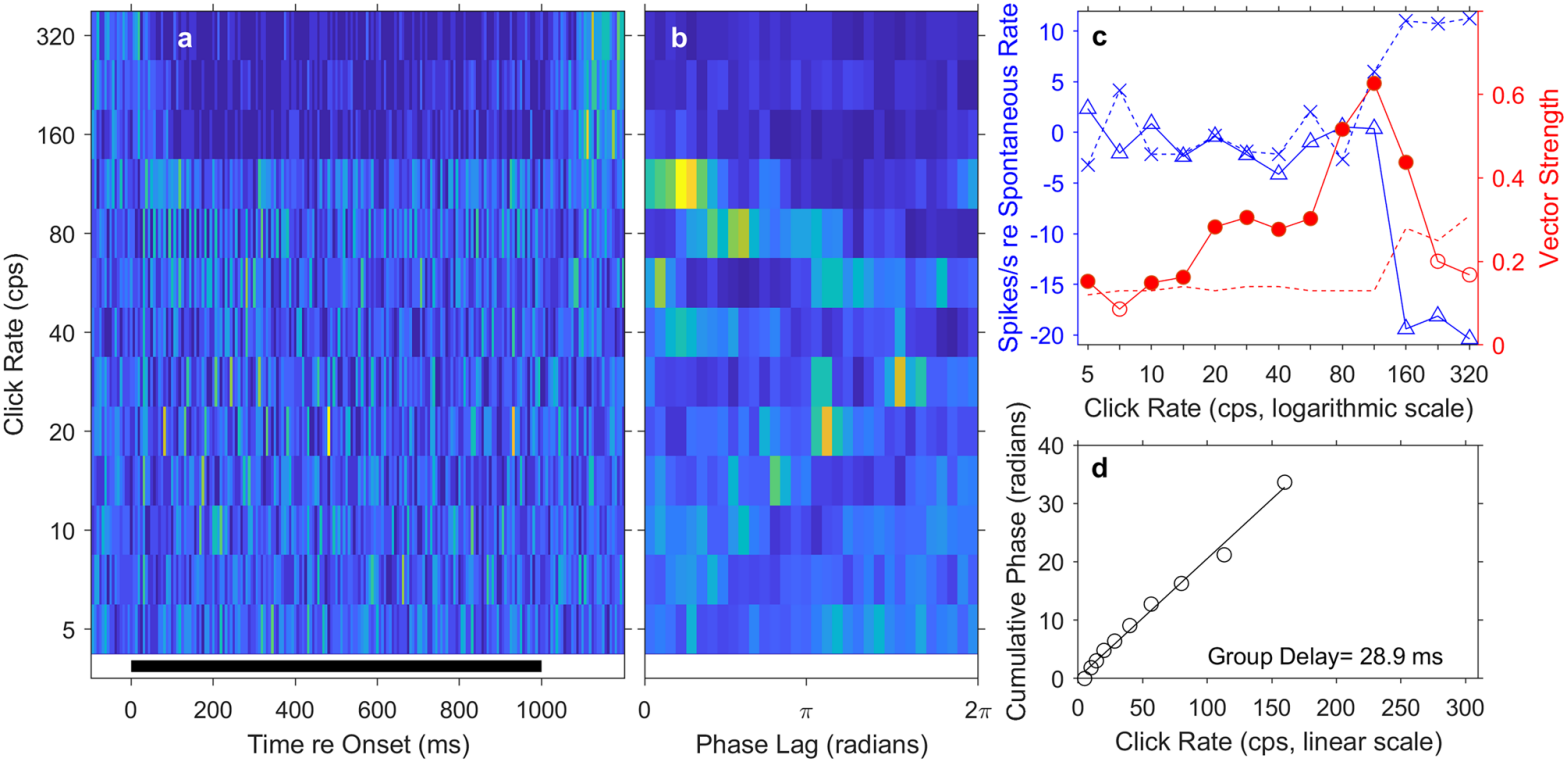
Temporal response of one unit, in this case showing suppression and offset responses to high click rates. Format of the 4 panels is identical to that in Fig. 7. In addition, the dashed blue line with X signs in panel **c** indicates mean rates of the offset responses (minus the mean spontaneous rate) as a function of click rate; the offset rate was computed over 10 to 190 ms after offset of the click trains. The values of those mean rates are given on the left axis

Many of the units responded to high click rates with suppression of tonic spike rates during the click train and/or with bursts of spikes (offset responses) after the offset of the click train. The unit presented previously in Fig. 8, for example, maintained a spike rate near the spontaneous rate at click rates up to 28 cps, at which point the tonic spike rate became strongly suppressed. Figure 10 shows an example of a multi-unit site for which the mean tonic rate (solid blue curve in Fig. 10(c)) remained near the spontaneous rate at click rates up to 113 cps. At higher click rates, then, the mean tonic spike rate was strongly suppressed. Of the 150 units that responded in any way to click trains, 70 responded to the highest click rate (320 cps) with tonic firing of suppressed spike rates that were significantly lower than the spontaneous spike rate (*P* < .05 after Bonferroni correction for 150 comparisons). The unit in Fig. 10 also exhibited a robust response to the offset of click trains at high rates, evident in the PSTHs in Fig. 10(a). The magnitude of the offset response is indicated in Fig. 10(c) by the dashed blue line and X symbols; the values of the offset rates are quantified on the left axis as mean spikes/s minus the spontaneous rate, the same as were the tonic responses during the click trains. Of the 150 units that responded to the click-train stimuli, 26 responded to the offsets of the 320-cps click trains with rates that were significantly higher than the rates of tonic responses during the click train (*P* < .05). Twenty-one units showed both significant suppression of tonic responses and significant offset responses, as in the example in Fig. 10(c).

#### Representation of Click Rates by Spike Rates of Non-synchronized Tonic Responses

**Fig. 11:**
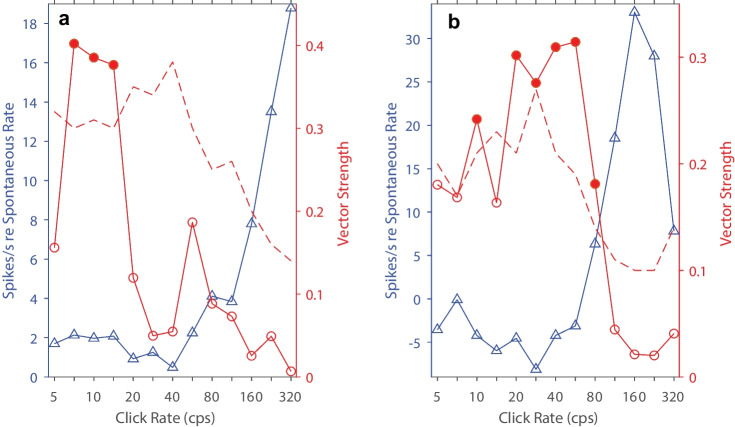
Summary measures of temporal responses of two units. Format of the 2 panels is identical to that in Fig. 7(c). **a** This unit showed synchronized responses to click rates of 7.1, 10, and 14.4 cps and non-synchronized monotonically-increasing rate responses to click rates > 40 cps. **b** This unit showed synchronized responses to most click rates up to 80 cps and non-monotonic rate response to click rates ≥ 57 cps. In this case, the spike rate peaked at 160 cps and declined at higher click rates

Many units (46/150) failed to synchronize to click rates higher than ~ 50 cps but represented click rates by their tonic spike rates. That is, they showed a non-synchronized tonic response that increased significantly in spike rate over a range of increasing click rates. An example of this type of unit is shown in Fig. 11a. Synchrony to the click trains (plotted by the red line) was evident at low click rates, from 7 to 14 cps, but synchrony was absent at 5 cps and at click rates > 14 cps. Nevertheless, at click rates beyond 40 cps, tonic spike rates (blue line) increased monotonically with increasing click rate to the highest click rate presented. This unit was representative of a common pattern that consisted of synchronized firing to low click rates and non-synchronized spike-rate representation of high click rates; such dual representation was observed in 20 of the 46 units that showed representation of click rates by their tonic spike rates.

Some of the units that showed representation by spike rates of non-synchronized tonic responses displayed a non-monotonic dependence on click rate. Figure 11b illustrates one such unit. The spike rate of this unit was at or below the spontaneous rate for click rates up to 57 cps. At higher click rates, spike rates increased sharply to peak at a click rate of 160 cps, then declined to 26% of the unit’s maximum spike rate at a click rate of 320 cps. This unit also showed synchronous firing at click rates from 20 to 80 cps. Of the 46 units that showed non-synchronized representation, spike rates of 30 units peaked at click rates < 320 cps and, at 320 cps, declined to an average of 36% of their highest spike rates; 12 of those 30 units also synchronized to low click rates.

**Fig. 12:**
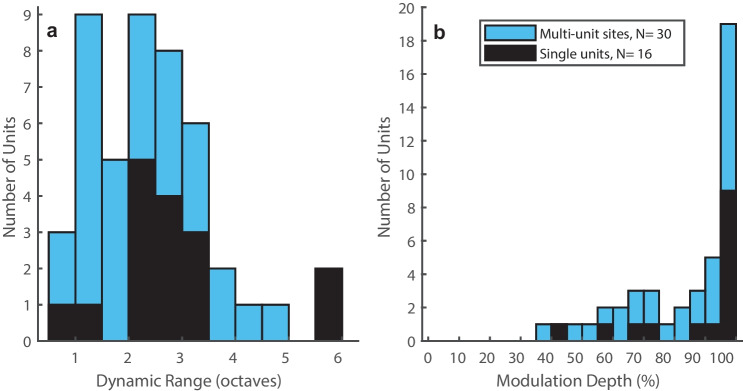
(**a**) Distribution of dynamic ranges of non-synchronized rate responses. All spike rates were computed in the range of 110–1010 ms after the onset of the click trains. Blue and black fill indicate multi- and single-unit responses, respectively. (**b**) Distribution of the depths of modulation of spike rates by varying click rates. The modulation depth was given by the maximum minus the minimum spike rates within the dynamic range of click rates as a percentage of the maximum minus minimum spike rates across all click rates

We observed a wide variation in the ranges of click rates that elicited non-synchronized rate coding by individual units. The dynamic range of non-synchronized rate coding for each unit was given by the range of click rates over which increases in click rates resulted in significant increases in unit spike rates. The units shown in Fig. 11 both had dynamic ranges of 2.5 oct, which was around the mode of the distribution of our sample, whereas some other units had dynamic ranges of ≥ 4 oct; the median across all 46 units was 2 oct. Figure 12(a) plots the distribution of dynamic ranges for non-synchronous coding by multi-unit sites (in blue; median = 2.0 oct, *N* = 30) and by well-isolated single units (in black; median 2.5 oct, *N* = 16); the distributions of single- and multi-unit dynamic ranges were not significantly different (*ks2stat* = 0.24, *P* = 0.46). For most units that showed non-synchronized spike-rate representation of click rates, variation in click rate had a profound effect on their spike rates. The percentage ranges by which spike rates were modulated by variation in click rates are plotted in Fig. 12(b). Median spike-rate modulation depths were 92.4% for single units and 98.6% for multi-unit sites; the distributions of single- and multi-unit modulation depths were not significantly different (*ks2stat* = .20, *P* = .67).

**Fig. 13:**
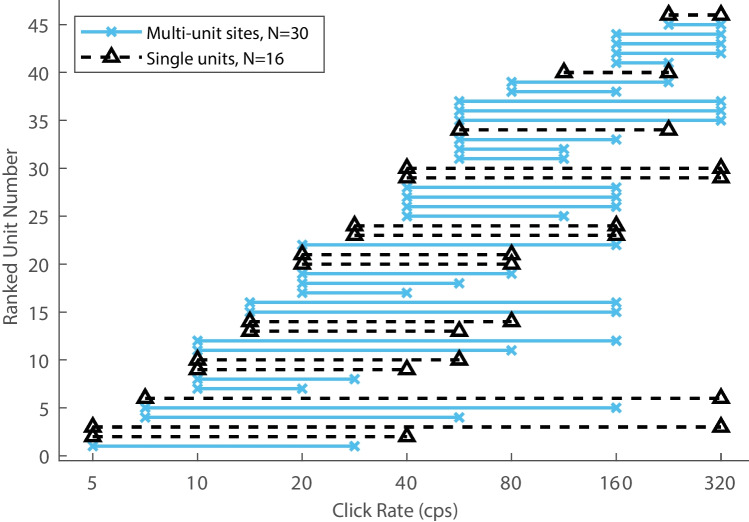
Dynamic ranges of single units. Each horizontal bar depicts the dynamic range of one unit, either a multi-unit site (blue bars) or a well-isolated single unit (dashed black bars)

Each of the click rates tested from 5 to 320 cps fell within the dynamic ranges of 3 or more of the 46 units that represented click rates by their spike rates. That is illustrated in Fig. 13, in which each horizontal line represents the dynamic range of one unit. A vertical line drawn at any click rate from 5 to 320 cps would cross 3 or more of the lines; the number is larger, 12 or more units, across the range of 10 to 320 cps. That indicates that all of those click rates could be represented by the click-rate-dependent spike rates of units in cortical area A1 of alert cats.

## Discussion

Recordings from cortical area A1 of alert cats reveal high-acuity features of spectral and temporal representation that have received only scattered mention in studies of anesthetized cats and other species. They have been especially highlighted, however, in studies of area A1 of the awake marmoset.

### Spectral Representation

Our study of responses to tones follows the work by Sadagopan and Wang [15] that was conducted in area A1 of awake marmosets. As in our results, those investigators found units having V-, I-, and O-shaped FRAs, some of which had sustained responses that narrowed to around units’ best frequencies. The O and V/I units formed, respectively, 64% and 36% of that sample in the marmoset compared to 25% and 75%, respectively, in our sample in the cat (Table 1). The higher percentage of O units in the marmoset might represent differences in adaptations for the ethological needs of the two species. Alternatively, we might have encountered more O units in the cat had we sought the medio-lateral region of A1 in which non-monotonic responses are more prevalent (as described below). The O pattern of restricted frequency and level tuning was first observed in the cat brainstem in unanesthetized, decerebrate conditions. Evans and Nelson [39] and Young and Brownell [40] described such units (“Type IV units”) in the cat’s dorsal cochlear nucleus. In a similar decerebrate preparation, Ramachandran et al. [31] observed V-, I-, and O-shaped FRAs in the cat’s inferior colliculus. In the cat inferior colliculus, low thresholds of O units coupled with their non-monotonic level sensitivity imply that their best levels generally were lower than those of V units, which agrees with our measurements of best levels in cortical area A1 in the alert cat. That contrasts with the broader distribution of best levels of O units in the marmoset cortex [15].

Sadagopan and Wang [15] emphasized the novelty of the joint spectral and level sensitivity of the O units in alert marmosets. Indeed, previous studies of A1 in anesthetized cats have not characterized spectral and level sensitivity of FRAs together in such quantitative detail. Nevertheless, there have been accounts of narrow, level-insensitive frequency selectivity and of non-monotonic level selectivity in area A1 of anesthetized cats. Phillips et al. [17, 41] reported that nearly half of their units were strongly non-monotonic and displayed response areas that were often completely circumscribed, like the FRAs of O units. Similarly, Sutter [42] reported that 20% of his sample had circumscribed FRAs. In anesthetized cats, circumscribed FRAs are more prevalent in the posterior field than in area A1 [43]. Circumscribed FRAs have been reported in area A1 of anesthetized ferrets but, as in cats, such units are more common in fields posterior to area A1 [44].

Regardless of FRA shapes, non-monotonic rate-level responses in anesthetized auditory cortex are widespread across species. Examples include mustached bat [45], mouse [46], rat [47], gerbil [48], chinchilla [49], ferret [44], and owl monkey [50]. In anesthetized cats, the prevalence of non-monotonic responses varies along the dorso-ventral dimension of area A1 [51]. Sutter and Schreiner [20] found a high concentration of non-monotonic units in a central region of A1 that appeared to coincide with a region of sharp frequency tuning [27, 52]. One might speculate, then, that many neurons in that central region would exhibit O-shaped FRAs even in anesthetized conditions. We note that, contrary to the work from the Schreiner lab in anesthetized cats and our work in alert cats (Fig. 3), the coincidence of sharp frequency tuning and non-monotonic responses was rather variable across anesthetized cats in a study by Heil et al. [53]. We observed no topographic concentration of O units in our study of alert cats, but our experimental design was not well suited for such mapping.

Our results obtained with single- versus multiple-unit recordings showed minimal or no differences in bandwidths or monotonicity indices. The absence of a strong sensitivity to unit isolation suggests that neighboring neurons tend to share similar frequency and level sensitivity. That indicates, in turn, that multi-unit recording would not preclude observation of O-shaped FRAs. Previous studies in anesthetized cats have demonstrated local similarities in monotonicity [51, 54, 55] and in sharpness of frequency tuning (e.g., [34, 52]). Notably, however, Sutter and Schreiner reported effects of unit isolation on non-monotonicity [20] and on bandwidths [34]. Those unit-isolation effects in anesthetized cats contrast with results obtained in our awake preparation.

Studies of area A1 in the awake marmoset have highlighted the sharpness of frequency tuning. In marmosets, the mean bandwidth at best level was 0.43 oct across all units [14] or 0.25 oct across just the O units [15]. Our results in the alert cat compared favorably: our median bandwidth across all units was 0.47 oct (0.34 oct for units with BFs within 1 oct of 8 kHz), and the median across just the O units was 0.22 oct. Bandwidth values from awake and anesthetized marmoset and cat studies are given in Table 2.

In anesthetized cats, frequency bandwidths in A1 vary widely across conditions of anesthesia, but generally are broader than those in alert conditions. Under barbiturate, mean bandwidths at 40 dB above threshold were as low as 0.31 oct within just the sharply tuned central area of A1 [27], but bandwidths at only 20 dB above threshold could average 0.60 oct across all units [1]. Frequency tuning was much broader under halothane, an inhalation anesthetic, than in previous results obtained with barbiturate: bandwidths under halothane averaged 4 oct at 40 dB above threshold [28]. Cheung et al. [56] characterized responses in cat area A1 under a different inhalation anesthetic, isoflurane, and then tested the same recording sites in the same animals after administration of barbiturate (and cessation of isoflurane). In that study, there were no significant differences in characteristic frequencies or in bandwidths between anesthetic regimes, although thresholds were 12 dB higher and first-spike latencies were 2 ms longer under isoflurane.

Gaese and Ostwald [57] recorded from area A1 in rats with chronically implanted electrodes. Unit activity was studied under awake conditions, and then light anesthesia was induced with Equithesin (containing chloral hydrate and sodium pentobarbital). Some 30–45 min after Equithesin injection, 71% of units lost their FRAs. Moreover, many of the remaining FRAs reduced in size, giving the appearance of sharper frequency tuning. That result conflicts with our observations that frequency tuning in awake cats tends to be sharper than is reported in anesthetized conditions.

In our sample, units having the narrowest bandwidths tended to respond rather exclusively to low sound levels, and units that responded best to higher sound levels tended to have broader bandwidths; similar trends were reported in awake marmosets [15]. That means that cortical responses in aggregate show broader frequency tuning with increasing sound levels both because (1) the tuning of single units, at least of V units, tends to increase with increasing levels, and (2) neurons having high best levels tend to have broader tuning. Perceptual measures of frequency selectivity in humans using notched-noise techniques also show a broadening of perceptual auditory filters associated with increasing signal levels (e.g., [58, 59]). In those studies, equivalent rectangular bandwidths at 4 kHz, roughly equivalent to the 8-kHz audibility region in cats, increased by ~ 10% between signal levels of 30 and 70 dB SPL [58]. As in our cortical results, perceptual auditory filters tend to expand asymmetrically, toward lower frequencies. Those characteristics of perceptual auditory filter shapes are captured well by non-linear cochlear models. The broadening of FRAs of cortical V units that we observed, along with the low-frequency asymmetry, agrees at least qualitatively both with perceptual results and with cochlear models, suggesting that the 6 or more levels of synaptic integration from ear to cortex have minimal effect on the frequency selectivity for steady-state sounds. The level sensitivity of O units and the level-insensitive frequency tuning of I units, however, present examples of transformations that can occur within those 6-plus synaptic levels.

### Temporal Representation

Representation of click trains by explicit phase locking of cortical units is limited to low rates in anesthetized conditions. One previous study in anesthetized cats showed median synchronization boundaries as high as 25 cps [12], but most studies show median synchronization boundaries ≤ 15 cps (e.g., clicks: [10, 11]). Synchronization boundaries extend to higher rates in awake conditions. Phase locking at rates ≥ 100 cps was reported in an early study of unanesthetized, muscle-relaxed, cats [60]. Dong et al. [61] found in awake cats that about 21% of the single units synchronized to a 50-cps click rate; that compares with our observation that 22% of synchronized units had synchronization boundaries ≥ 50 cps. In awake marmosets [18], only 36 of 190 click-responsive units (19%) showed synchronized responses. Among just those synchronized units, the median synchronization boundary was ~ 47 cps. In the present study, 72 of 150 click-responsive units (48%) synchronized to 5 cps or higher. The median synchronization boundary of those synchronized units was 30 cps, with 9 units synchronized to rates ≥ 80 cps. The phase-locked representation of click rates in our alert preparation accords with that in previous unanesthetized preparations and further supports the idea that cortical neurons synchronize to higher click rates in awake compared to anesthetized conditions.

In awake marmosets, Lu et al. [18] observed that 50 of the 190 click-responsive units were not synchronized to clicks but represented increasing click rates by increasing spike rates; these were “non-synchronized rate responses.” An additional 8 units showed “mixed” synchronized and non-synchronized responses. Half of the non-synchronized rate responses in the marmoset study were restricted to click ranges > 78 cps. In our study in alert cats, 46 of 150 click-responsive units showed tonic spike rates that increased significantly with increases in click rate. Our results differed from those in marmosets in that dynamic ranges of click-rate representation by spike rates covered nearly the entire tested range of click rates. That means that most of our tested range of click rates could be represented in area A1 both by explicit spike synchrony and by spike rates of non-synchronized tonic responses, in some cases by units that showed both synchronized responses to low click rates and non-synchronized rate responses to higher click rates.

We acknowledge that variation in click rates was somewhat confounded by variation in sound level. That is, our minimum ½-oct increments in click rates were accompanied by ~ 1.5-dB increases in sound level due to the increase in the number of clicks per unit time, and 2-oct increments in click rates (the median dynamic range of non-synchronized rate representation) were accompanied by ~ 6-dB increases in sound level. Those small increments in sound level might have contributed to the increases in neural spike rate that was often associated with increasing click rates. We think, however, that increases in cortical spike rates due to level increases would have been small compared to the increases in spike rates that we observed in response to increases in click rate, typically close to 100% modulation of firing rates across 2-oct changes in click rates. In the study in awake marmosets [18], similar non-synchronized rate responses and stimulus-synchronized responses whether or not sound levels were adjusted for changes in click rates.

We are interested in how cortical representation of the rates of clicks and other repetitive sounds might support perceptual discrimination of temporal, non-spectral, pitch (see [62]). In a recent psychophysical study, cats discriminated the rates of bandpass-filtered pulse trains, with a passband centered on 8 kHz [63]. Cats could readily detect 36 or 66% increases in pulse rates above pulse rates of 188 to 560 pulses per second (pps); performance was degraded for base rates of 94 pps. The range of pulse rates 188 pps and higher was above the synchronization boundary of > 90% of all the synchronized units in the present cortical study. That suggests that psychophysical pulse-rate discrimination does not require synchronized firing of neurons in area A1. Indeed, most of the range of rates to which neurons synchronized in our study coincides with the range of repetitive sounds that human listeners report as “flutter” rather than pitch [64]. Nevertheless, the range of robust psychophysical pulse-rate discrimination in the Richardson study, 188 to 560 pps, was well covered by the dynamic ranges of non-synchronized spike-rate representation in the present study, at least up to our highest-tested rate of 320 cps.

In the study by Richardson et al. [63], pulse-rate changes in the range of robust psychophysical performance produced a scalp-recorded potential, the auditory change complex (ACC), which is thought to originate from cortical generators (e.g., [65]). Increments in pulse rates would have produced increments in spike rates in non-synchronized neurons like those observed in the present study. We speculate that such increments in cortical unit activity might appear on the scalp as ACCs and could underlie psychophysical discrimination of temporal pitch changes.

### Conclusions

Our measures of spectral and temporal representation in area A1 of alert cats compare well with those reported from awake marmosets [14, 15, 18]; notable differences in the cat include the smaller percentage of O units and the broader range of click rates represented by spike rates of non-synchronized units. That comparison indicates that sharp spectral and temporal acuity are not limited to primates and, indeed, might be widespread among mammals. Our minimal differences in results between single- or multi-unit recording refute the hypothesis that some previous failures to observe sharp spectral and temporal acuity were due to the use of multi-unit recording. It now seems clear that the principal factor accounting for differences between the awake-marmoset results and previous results from anesthetized cats is awake-versus-anesthetized conditions.

Measures of aggregate spectral sensitivity across our sample of A1 in awake cats generally agree with perceptual measures of auditory filters in that FRAs broadened with increasing sound levels, primarily toward low frequencies. The perceptual significance of the variety of O-, I-, and V-shaped FRAs, however, remains unknown. Our temporal measures highlight the importance of non-synchronized rate coding by spike rates across the range of stimulus rates relevant to temporal pitch perception. Mechanisms for conversion of phase-locked codes at lower-brainstem levels to rate codes in the cortex are not well understood. These topics of spectral and temporal representation in the cortex remain for future research.
